# Older People’s External Residential Assessment Tool (OPERAT): a complementary participatory and metric approach to the development of an observational environmental measure

**DOI:** 10.1186/s12889-016-3681-x

**Published:** 2016-09-29

**Authors:** Vanessa Burholt, Matthew Steven Roberts, Charles Brian Alexander Musselwhite

**Affiliations:** Centre for Innovative Ageing, College of Human and Health Science, Swansea University, Singleton Park, Swansea, SA2 8PP UK

**Keywords:** Environment, Residence characteristics, Neighbourhood, Community, Domicile, Aged, Environment and public health, Environment design, Environment policy

## Abstract

**Background:**

The potential for environmental interventions to improve health and wellbeing has assumed particular importance in the face of unprecedented population ageing. However, presently observational environmental assessment tools are unsuitable for ‘all ages’. This article describes the development of the *Older People’s External Residential Assessment Tool (OPERAT)*.

**Methods:**

Potential items were identified through review and consultation with an Expert Advisory Group. Items were ranked according the importance ascribed to them by older people who responded to a survey distributed by 50+ forum in Wales (*N =* 545). 40 highly ranked items were selected for the OPERAT pilot. An observational assessment was conducted in 405 postcodes in Wales. Items validated with data from a survey of older residents (*N =* 500) in the postcode areas were selected for statistical modelling (Kendall’s *Tau-b, p* < .05). Data reduction techniques (exploratory factor analysis with Geomin rotation) identified the underlying factor structure of OPERAT. Items were weighted (Thurstone scaling approach) and scores calculated for each domain. Internal consistency: all items were tested for scale-domain total correlation (Spearman’s rank). Construct validity: correlation analysis examined the associations between domains and the extent to which participants enjoyed living in the area, felt that it was a desirable place to live, or felt safe at night or during the day (Spearman’s rank). Usability: analysis of variance compared mean OPERAT domain scores between neighbourhoods that were homogenous in terms of (a) deprivation (quintiles of the Townsend Index) and (b) geographic settlement type. Inter-rater reliability: Krippendorff’s alpha was used to evaluate inter-rater consistency in ten postcode areas.

**Results:**

A four factor model was selected as the best interpretable fit to the data. The domains were named *Natural Elements, Incivilities and Nuisance; Navigation and Mobility;* and *Territorial Functioning.* Statistical tests demonstrated good internal consistency, convergent validity, utility and inter-rater reliability.

**Conclusions:**

Participatory approaches to research and robust statistical testing are not mutually exclusive. OPERAT can be used to assess the suitability of external residential environments for older people with different physical and cognitive capacities, living in rural or urban areas. OPERAT can be used to help plan residential environments that are friendly for all ages.

**Electronic supplementary material:**

The online version of this article (doi:10.1186/s12889-016-3681-x) contains supplementary material, which is available to authorized users.

## Background

### The importance of the environment to older people

The Faculty of Public Health in the UK defines public health as “the science and art of promoting and protecting health and wellbeing, preventing ill health and prolonging life through the organised efforts of society” [1]. Throughout this article wellbeing is defined as the instances “when individuals have the psychological, social and physical resources they need to meet a particular psychological, social and/or physical challenge” [2]. The potential for environmental interventions to improve public health (mortality, chronic conditions, mental health, and health behaviours) and wellbeing outcomes for residents [3] has assumed particular importance in the face of unprecedented global population ageing. In the 27 states of the European Union, the proportion of people aged 65 years or older will increase from 16 % in 2010 to 24 % in 2035. Similarly, the UK population is projected to become more aged with the proportion of people aged 65 years or older increasing from 17 % in 2010 to 23 % by 2035 [4]. The growth of this segment of the population has resulted in specific housing and community needs. The preference of many older people to ‘age in place’ has led to public policies supporting community living [5]. Developing age-friendly and dementia supportive communities is considered as one of the most effective local policy approaches for responding to demographic ageing [6].

The physical environment plays a substantial role in maintaining ‘spatial independence’ (the freedom and choice to access public physical space). This may be particularly salient for older people with physical or cognitive impairment who may encounter barriers to spatial independence [5]. The correspondence between functional or cognitive ability and environmental press (or the demands of the environment) is called person-environment fit [7].

Between one third and one-half of older adults experience some form of mobility impairment or limitation [8]. Furthermore, cognitive impairment and the risk of dementia increase with age. The prevalence of dementia in the English population aged 65 years or more, is estimated to be around 6.5 % (670,000 people) [9]. It is important to have robust methods to identify environmental deficits that exclude marginalised groups (such as people with dementia) so that local planners, local authorities and other organisations can plan interventions to improve health and wellbeing outcomes for older residents and meet public health goals.

### Environmental influences on health and wellbeing

The influence of the external environment on health and wellbeing is well established. There are three main theories that attempt to explain the link between observable environmental features and personal health and wellbeing outcomes: *environmental aesthetics, environmental stress* and the *neighbourhood disorder* model [10].

*Environmental aesthetics* refers to the beauty of natural elements of the environment. Natural environments can influence psychological wellbeing, by decreasing tension, anger and depression and have restorative benefits [7]. Kaplan and Kaplan [11] suggest that humans have an automatic affective biological response to natural landscapes which influences positive outcomes, also referred to as biophilia [12].

Neighbourhood design, housing diversity, population density, mixed land use and open space are associated with physical activity such as walking and intentional and spontaneous social contact [13–17]. In these instances, it is assumed that physical and social activity within a neighbourhood are moderated by an individual’s ability to cope with *environmental stress* or hazards. Crime and fear of crime may also reduce physical and social activity and these are influenced by *neighbourhood disorders* such as litter, graffiti, land use, lighting and housing quality [18]. The impact of both *environmental stress* and *neighbourhood disorders* are important as lower levels of physical activity and social isolation are associated with poor health outcomes.

### Observational environmental assessment tools

There is a vast body of evidence on observational environmental assessment tools which has been well-summarised in review articles (e.g. [19–21]). Here, we summarise the constructs or domains that most observational tools purport to address, that is ‘defensible space’, ‘natural environment’, ‘territorial functioning’, ‘physical incivilities’, ‘land use’ and ‘accessibility and safety from traffic’.

*Defensible Space* refers to elements of the environment which encourage residents to exert territorial control such as walls and symbolic barriers like gardens or shrubberies [22]. Tools measuring defensible space may capture incidences of security bars or grates on windows (e.g. Project on Human Development in Chicago Neighbourhoods [23]; Block Environmental Inventory [24]), or hedges and gates in front of properties (e.g. Residential External Assessment Tool (REAT) [25]).

*Natural Environment* includes greenery and landscaping. Tools assessing elements of the natural environments may count the number of trees in a given area (e.g. REAT [25]); or the presence of fields, lakes, forests or the ocean (e.g. Irvine-Minnesota Inventory to Measure Built Environment [26]).

*Territorial Functioning* relates to the personal investment that residents have in an area [27]. Tools assessing territorial functioning capture how well private spaces are maintained [28] and may include items that assess the quality of residential upkeep (e.g. Irvine-Minnesota Inventory [26]) or garden maintenance (e.g. REAT [25]).

*Physical Incivilities* refers to instances of neglect and deterioration in an area. Graffiti, vandalism and littering may signal declining quality of life in a neighbourhood [29]. Tools that capture physical incivilities count the occurrences of dog fouling, litter (e.g. Healthy Environments Partnership Neighbourhood Observational Checklist (NOC) [21]) used condoms (e.g. Neighbourhood Inventory for Environmental Typology (NIfETY) [30]) or abandoned or vacant properties in the area (e.g. REAT [25]; NIfETY [30]).

*Land use* refers to the functional use of the land or buildings. Non-residential land use is considered in terms of the opportunities for crime [30] or in relation to the vibrancy and vitality of an area [31]. Tools capturing land use may record the number or purpose of commercial properties such as bars, pawn shops (e.g. NOC [21]; NIfETY [30]), cafes or galleries (e.g. Irvine-Minnesota Inventory [26]).

*Accessibility and safety from traffic* addresses the extent to which people can traverse the neighbourhood. It takes into account hazards presented by poorly maintained roads or pavements and traffic. Some tools consider the incline, maintenance and width of pavements and/or roads (e.g. Irvine-Minnesota Inventory [26]), or look for evidence of cycle paths, traffic calming measures and pedestrian crossings (e.g. Systematic Pedestrian and Cycling Environmental Scan (SPACES) [31]).

There is a range of observational environmental assessment tools available. However, they have shortfalls that undermine their suitability for ‘all ages’. Firstly, a limited number of studies have examined the psychometric properties of the observational scales, made use of data reduction techniques, or reported inter-rater reliability (see Schaefer-McDaniel et al. [20] for an overview). Secondly, most tools have been developed and used in urban and suburban areas and items more pertinent in rural areas (e.g. an industrial agricultural outlook) are absent [20]. Thirdly, very few tools have been designed with the needs of older people in mind. For example, REAT excluded older people (aged 75 years or more) from the development stage [25]. Although the Neighbourhood Design Characteristics Checklist (NeDeCC) involved older people in its design, researchers had an a priori conceptualisation of the underlying constructs and selected the items for the tool [32]. In omitting or restricting the input of older people during development, particular environmental needs may have been overlooked: REAT does not consider *accessibility* and NeDeCC omits *territorial functioning*.

The purpose of this article is to describe the development of the *Older People’s External Residential Assessment Tool (OPERAT)* and to examine data quality, reliability and construct validity of the tool*.* OPERAT is an observational tool specifically designed to assess the suitability of external residential environments for older people (aged 65+ years) with different physical and cognitive capacities, living in rural or urban areas. We explain how we:

1. Identified items for OPERAT specific to older people’s needs through consultation with an Expert Advisory Group and a postal survey with members of 50+ forum in Wales, UK.
2. Conducted an observational assessment using a pilot version of OPERAT in 405 postcodes in Wales.
3. Used data reduction techniques (with items validated by a survey of older residents) to identify the underlying factor structure of OPERAT.
4. Weighted each of the items and domains in OPERAT according to the importance ascribed to them by older people.
5. Tested OPERAT for internal consistency, construct validity, usability and inter-rater reliability.

## Methods

### Development of the tool

A review of external residential measures generated a list of items spanning the six domains noted in the introduction. The items and domains were presented to a focus group (*N* = 15)—the Expert Advisory Group (EAG)—comprising people aged over 65 years, and representatives from the Royal National Institute for the Blind. Volunteers were recruited from Ageing Well in Wales’s networks [33] and were brought together for the purpose of the research. The participants responded to a request for volunteers with particular knowledge, interest (e.g. retired planner) or specific insights into pertinent environmental issues (e.g. from the perceptive of disability or visual impairment). Separate consultations with people with dementia and their carers (*N* = 4) were undertaken. This egalitarian method allowed for free-flow of discussion and debate to ensure that topics were covered that may be absent from extant (academic) accounts of environmental features, and allowed participants to develop subjects that were most important to them. Through a thematic analysis of discussion notes [34], key themes were identified which informed a final list of 84 items.

The Thurstone scaling approach was used to solicit ratings (magnitude and direction) from older people for each of the 84 items [35, 36]. Approximately 3200 weighting questionnaire were sent to thirteen 50+ forums in Wales for distribution to members [37]. For each item older people were asked to respond to the following questions:

- Is this a feature of your street? (Yes or no).
- Is/would this be good or bad feature? (Positive or negative).
- How much would/does each feature affect your satisfaction with the area? (Not at all; very little; a little; quite a lot or a great deal)*.*

Responses to the survey items were ranked by mean scores (for question (c)). The highest ranked items were used to develop 40 area assessments questions. For weightings, scores were transformed to a 9 point scale ranging from ‘a great deal (unfavourably)’ to ‘a great deal (favourably)’. The mid-point of the scale (5) was ‘not at all’. Items were omitted from analyses when there was no clear consensus about the positive or negative attributes of the feature.

### Creating the composite score

Observational and validation data were collected in 405 post code areas in Wales purposively selected for socio-economic and geographical diversity and a high proportion of older residents (>20 %) (Table 1). Area assessments were conducted by a single researcher who rated each of the 40 items while walking through the postcode area. The length of assessments ranged from 1 to 35 (*M* = 8.82, SD 5.93) minutes depending upon the size of the postcode.

**Table 1:** Distribution of assessments by tertiles of Welsh Index of Multiple Deprivation [67] and rural/urban classification

WIMD:	Least deprivation % (n)	Average deprivation % (n)	Most deprivation % (n)	*Total* *% (n)*
City and Town	13.3 (54)	13.3 (54)	13.3 (54)	*40 (162)*
Rural Town & Fringe	6.6 (27)	6.6 (27)	6.6 (27)	*20 (81)*
All others (rural)	13.3 (54)	13.3 (54)	13.3 (54)	*40 (162)*
*Total*	*33.3 (135)*	*33.3 (135)*	*33.3 (135)*	*100 (405)*

A validation questionnaire was distributed to every household (*N* = 9000) in the 405 postcode areas. The questionnaire stipulated that only one resident in the household aged 65 years or more should complete the survey. To enable a comparison between the OPERAT assessment and residents’ perceptions of the area, the validation questionnaire comprised items which corresponded to OPERAT items [38]. Responses for each item ranged from strongly agree (1) to strongly disagree (6). Bivariate correlation analyses (using Kendall’s *Tau-b*) examined the relationship between residents’ perceptions of their area and the corresponding OPERAT items. A significance level of *p* < 0.05 was applied to the tests.

There was potential for non-responder bias in the validation self-completion questionnaire, for example underreporting by frail, cognitively or visually impaired older people [39–41]. Thus, the EAG was consulted about items to test in exploratory factor analysis (EFA). Subsequently, some items were modified (combined and/or recoded) and others that were not significantly correlated with the validation questions were retained.

Items that captured the percentage of properties with a particular element (i.e. external beautification, trees in the garden, well maintained garden, well maintained property) were retained in their original form. All other variables were transformed so that 0 represented the most desirable state and 1 represented the less desirable state.

Twenty-three variables were tested with EFA. Principal axis factoring with Geomin rotation was used to determine whether there was an underlying factor structure which captured different domains of the external environment. Eigenvalues of one or more [42], scree plots and model fit indices (Chi square statistic; comparative fit indices (CFI); Tucker Lewis Index (TLI) and root mean square error of approximation (RMSEA)) were used to determine the number of factors which explained variation in the model. Steps were taken to refine the model. In total, seven variables were omitted on the basis of low factor loadings (<0.4). After each item deletion, the model was re-estimated.

The structure of the factor model was used to calculate domain scores and a global score for OPERAT. We determined the weight for each item identified in EFA from the distribution of ratings (see development of the tool). The raw weight of each item was the median of the distribution. For item ratings the midpoint of the scale (5) was subtracted from the raw weight. Items that had not previously been transformed for EFA were recoded in the same manner as the ‘count’ variables above. Next, Thurstone weights were used as multipliers in the composite OPERAT domain scores (the factors identified in EFA). Prior to summing the scores for each domain, items were multiplied by an integer between 1 and 4. Items assigned weights of +2 or -2 on the Thurstone scaling method were multiplied by 2; items assigned weights of +3 and -3 were multiplied by 3, and so on. The items in each domain were summed to produce a raw domain score.

The domains were rated in importance by the EAG, and weighted accordingly. The final score for each domain was computed as the transformed raw domain score. Each domain had the potential to comprise different number of items and different ranges of scores. Therefore, in order for the final OPERAT score to weight each domain according to the rank assigned by the EAG, the raw domain scores were transformed using the formula below.

$$ \begin{array}{l}\mathrm{Let}\ \upchi = 100/\mathrm{EAGweightF}1+\mathrm{EAGweightF}2+\dots .\ \mathrm{and}\ \mathrm{so}\ \mathrm{o}\mathrm{n}.\\ {}\mathrm{Transformed}\ \mathrm{domain} = \left.\frac{\left(\mathrm{Actual}\ \mathrm{r}\mathrm{aw}\ \mathrm{score}\ \hbox{--}\ \mathrm{lowest}\ \mathrm{possible}\ \mathrm{r}\mathrm{aw}\ \mathrm{score}\right)}{Possible\ \mathrm{r}\mathrm{aw}\ \mathrm{score}\ \mathrm{r}\mathrm{ange}}\right\}\kern0.5em *\ \left(\upchi\ *\ \mathrm{E}\mathrm{A}\mathrm{G}\ \mathrm{weight}\ \mathrm{f}\mathrm{o}\mathrm{r}\ \mathrm{the}\ \mathrm{domain}\right)\end{array} $$

The OPERAT score is a composite of weighted domains scores in which higher scores represent a less desirable state.

### Internal consistency, construct validity, usability and inter-rater reliability

All items were tested for scale-domain total correlation using Spearman’s rank correlation. A minimum correlation of 0.2 between any item and the item-domain total score justified inclusion in the domain [43].

Convergent validity was examined by estimating (Spearman’s rank) correlations between the observational OPERAT domains, residents’ perceptions of their neighbourhood and neighbourhood structural characteristics [38]. Residents’ perceptions of their neighbourhood were captured by items assessing the extent to which they enjoyed living in the area, felt that it was a desirable place to live, or felt safe at night or during the day. Responses for each item ranged from strongly agree (1) to strongly disagree (6). A composite measure of aesthetic attachment to place was also calculated as the transformed sum of items assessing the importance of scenery, space and peacefulness in creating sense of belonging to the area, where higher scores represent a greater attachment [7]. A significance level of *p* < 0.05 was applied to the tests.

The Townsend Index of Deprivation represents a neighbourhood structural characteristic [44]. It uses variables derived from the census on unemployment, overcrowded households; car/vans ownership and home ownership and is calculated for Lower Super Output Areas (LSOAs). OPERAT area assessments were linked to LSOAs by postcode to capture deprivation within each area.

We tested the utility of OPERAT by examining the variability of the indicators within neighbourhoods that are relatively homogenous in terms of (a) deprivation and (b) geographic settlement type. Analysis of variance (ANOVA) was used to compare mean OPERAT domain scores between quintiles of the Townsend Index and between the three settlement types [45] used in the purposive selection of the postcode areas. Post hoc tests (Tukey) examined differences in mean scores between homogenous subsets.

Ten postcode areas were selected to assess inter-rater reliability. Two raters were trained to use OPERAT and assessment data were analysed using the SPSS macro KALPHA to establish Krippendorff’s alpha [46]. Krippendorff’s alpha has a range between 1 and 0 where 1 indicates perfect agreement. Inter-rater agreement was demonstrated by scores ≥0.8 [47].

## Results

A literature review and focus group discussions identified 84 items that could potentially contribute to OPERAT. These were included in a questionnaire that was distributed to older people through the 50+ forum in Wales. 545 older people returned the ‘weighting’ questionnaire. The mean age of respondents was 69.61 years (SD 9.16) and 63.5 % were female. The majority of respondents were married or living as a couple (52.4 %), with fewer participants reporting that they were widowed (22.9 %), divorced or separated (18.9 %), or never married (5.9 %). Fewer than one-third (31.9 %) of participants reported a long-term health condition limiting their ability to undertake daily activities.

Items that respondents ranked highest were selected for the OPERAT pilot assessment [see Additional file 1]. On advice from the EAG some lower ranked items were combined with higher ranked items to make composite assessment question. Forty items were included in the pilot OPERAT assessment in 405 postcode areas [see Additional file 2].

### Empirical construct validity

606 older people living in the 405 postcode assessment areas returned a ‘validation’ questionnaire, of which 500 were eligible for inclusion. 116 were excluded because the respondent did not provide a full postcode (*N* = 51); the postcode was not in an assessment area (*N* =45); the respondent was younger than 65 years (*N* =9); or no age was provided (*N* =11). This is an estimated response rate of 37 %, which is comparable to other postal surveys with older people [48]. The average age of the sample of 500 participants was 74.07 years (SD 7.79) and more than half (52.2 %) were male. A majority of participants were married or living with a partner (54.6 %), with fewer participants reporting that they were widowed (28.8 %), divorced or separated (13.6 %) or never married (3 %). More than one-third (38.6 %) reported a long-term health condition which limited their ability to undertake daily activities.

The correlation of responses to the validation survey with items in the OPERAT pilot tool indicated significant associations between 21 of the 40 items [see Additional file 2]. In addition to exploring the underlying factor structure of these items in EFA, the EAG recommended that other items should be retained because of their importance to people with dementia or visual impairment. Thus, items relating to the presence and readability of roads signs were collapsed to a single item (are there clear and easy to read road name signs?). Similarly, the item relating to walls or buildings blocking out light was retained, as were the items relating to street and alleyway lighting which were collapsed into a single item (are there lights on the streets and in alleyways?). For parsimony (and to reduce the likelihood of matrix errors) eight further items were collapsed into three new items for EFA. During EFA model refinement six variables were dropped from the analyses on the basis of low factor loadings (<0.4). The final model included 16 items.

Four factors were extracted with eigenvalues in excess of one. However, the scree plot supported a four or five factor solution. The fit statistics showed a good fit across all indices for the four-factor model, although the five-factor model had a slightly better fit (Table 2). The four-factor model was selected as the best interpretable fit to the data: in the five-factor solution one of the factors comprised just one item. The structure of the four-factor model is summarised in the Geomin rotated pattern matrix (Table 3).

**Table 2:** Fit indices for exploratory factor analysis

Model	*χ* ^2^	*p*	CFI	TLI	RMSEA
1 factor	1901.82	<0.001	0.72	0.68	0.21
2 factor	654.46	<0.001	0.91	0.88	0.13
3 factor	386.33	<0.001	0.95	0.92	0.10
4 factor	146.35	<0.001	0.98	0.98	0.06
5 factor	81.07	<0.01	0.99	0.99	0.04
6 factor	39.65	>0.05	1.00	1.00	0.01

**Table 3:** Geomin rotated factor matrix: four-factor solution

Factor Name	Item	F1	F2	F3	F4
Natural Elements	Public grass or verges	0.87			
	Sounds of nature	0.56			
	# private trees	−0.49			
Incivilities and Nuisance	Traffic, industrial or other noise		0.80		
	Litter, dog fouling, broken glass		0.57		
	# cars passing		0.80		
Navigation and Mobility	Legible road signs			0.58	
	Street and alleys lit			0.58	
	Pavement maintenance & width			0.73	
	Road maintenance			0.79	
	Pavement/road gradient			0.48	
Territorial Functioning	External beautification				0.49
	Nature of parking				−0.42
	Garden maintenance				0.89
	Property maintenance				0.63
	Industrial/commercial outlook				−0.79

We named Factor 1 *Natural Elements* as it comprised items relating to the presence or absence of public grass or verges; sounds of nature; and trees in gardens. Factor 2 was named *Incivilities and Nuisance* and comprised items relating to traffic, industrial or other loud noise; incidences of litter, dog fouling and broken glass; and volume of traffic passing. Factor 3 was entitled *Navigation and Mobility* and included five items assessing the legibility of road signs, lighting, and the quality of the pavement and road. We called Factor 4 *Territorial Functioning* as the five items related to the upkeep of properties and gardens, the nature of parking and whether the main outlook was (agricultural) industrial or commercial.

### Refinement of total and composite scoring

The EAG ranked the *Navigation and Mobilisation* domain as more important to older people than the other domains. Subsequently Factor 3 was assigned a weight of 2. Applying the formulas outlined in the methods the final domain scores were calculated as follows:

$$ \mathrm{Where}\ \upchi = 100/1+1+2+1 = 20 $$

Factor 1, *Natural Elements* was computed as:

$$ \left.\frac{\left(\mathrm{Raw}\ \mathrm{domain}\ \mathrm{score} - 0\right)}{9}\right\}\kern0.5em *\ 20 $$

Factor 2, *Incivilities and Nuisance* was computed as:

$$ \left.\frac{\left(\mathrm{Raw}\ \mathrm{domain}\ \mathrm{score}\ \hbox{--}\ 0\right)}{10}\right\}\kern0.5em *\ 20 $$

Factor 3, *Navigation and Mobility* was computed as:

$$ \left.\frac{\left(\mathrm{Raw}\ \mathrm{domain}\ \mathrm{score} - 0\right)}{13}\right\}\ *\ 40 $$

Factor 4, *Territorial Functioning* was computed as:

$$ \left.\frac{\left(\mathrm{Raw}\ \mathrm{domain}\ \mathrm{score} - 0\right)}{14}\right\}\ *\ 20 $$

The final OPERAT score was calculated as a sum of the four domains. Table 4 shows a summary of the scores. The lowest mean scores were observed for *Incivilities and Nuisance*, while the highest mean scores were for the *Navigation and Mobility* domain (which would be expected given the weight ascribed to this factor). Item-domain total correlations were all greater than 0.2, indicating a degree of internal consistency.

**Table 4:** Summary statistics for OPERAT domains

Domain	Natural elements	Incivilities and nuisance	Navigation and mobility	Territorial functioning	Total
Max possible score (raw)	9	10	13	14	
Max possible score (transformed)	20	20	40	20	100
Observed range	0–20	0–20	0–40	0–20	7.69–79.35
Mean	7.21	5.74	17.05	8.00	37.99
Median	7.78	3.00	15.39	8.57	38.40
S.D	5.42	6.32	10.51	3.33	13.59

### Convergent validity, utility and inter-rater reliability

In order to establish convergent validity, correlation analysis examined the associations between OPERAT domains and residents’ perceptions of their neighbourhood capturing the extent to which participants enjoyed living in the area, felt that it was a desirable place to live, or felt safe at night or during the day. In each case, greater domain scores represented a less desirable state, and greater scores on residents’ perceptions represented stronger disagreement with the statement (Table 5).

**Table 5:** Convergent validity between OPERAT domains, residents’ perceptions of area and deprivation at postcode level

	Natural elements	Incivilities and nuisance	Navigation, and mobility	Territorial functioning
*Individual level (n = 500)*				
I enjoy living around here	0.01	0.06	−0.10*	0.11*
I think of this place as a desirable place to live	0.03	0.08	−0.10*	0.14*
I feel safe around here during the day	0.04	0.10*	−0.07	0.01
I feel safe around here at night	0.06	0.18**	−0.10*	0.06
Aesthetic attachment to place	−0.11*	−0.09	0.10*	−0.09*
*LSOA level (n = 396)*				
Townsend Index	0.22**	0.25**	−0.17**	0.67**

* p < .05 **p <.005

Spearman’s Rho revealed a weak but statistically significant relationship between the natural elements in an area and place attachment. Those with greater aesthetic attachment to place tended to reside in areas with more natural elements (*r*_s_ = -0.11, *p* < .05), although this accounted for only 1 % of the variance in scores. Moreover, areas with fewer natural elements were weakly, but significantly associated with greater levels of area deprivation (*r*_s_ = 0.22, *p* < .01) accounting for around 5 % of variance in scores.

Spearman’s Rho demonstrated a weak but statistically significant relationship between incivilities and nuisance in an area and perceptions of safety: older people living in areas with fewer incivilities and nuisance felt safer during the day (*r*_s_ = 0.1, *p* < .05) and at night (*r*_s_ = 0.18, *p* < .01) than those living in areas with a greater presence of these items. This association accounted for only 3 % of variance in scores. At the postcode level, areas with greater levels of incivilities and nuisance were weakly, but significantly associated with greater levels of area deprivation (*r*_s_ = 0.25, *p* < .01) accounting for around 5 % of variance in scores.

The non-parametric correlations revealed that residents’ ratings of area enjoyment, desirability and safety at night (but not during the day) were associated with elements of navigation and mobility. Counterintuitively, areas with more elements that could potentially hamper navigation and mobility tended to be associated with greater ratings of enjoyment (*r*_s_ = -0.10, *p* < .05) desirability (*r*_s_ = -0.10, *p* < .01) and feelings of safety at night (*r*_s_ = -0.10, *p* < .05) than areas with more elements that could potentially facilitate navigation and mobility. In each case, the association accounted for around only 1 % of variance in scores. At the postcode level, the analysis showed a significant (but weak) association between areas with high scores in this domain (more barriers to navigation and mobility) and lower area deprivation (*r*_s_ = -0.17, *p* < .01) and accounted for around 3 % of variation in scores. While other elements of the OPERAT assessment were positively associated with deprivation (less desirable aspects of the environment were associated with greater area deprivation), barriers to mobility and navigation more frequently occurred in more affluent and less deprived areas.

The analyses demonstrated that territorial functioning was weakly associated with area enjoyment (*r*_s_ = 0.11, *p* < .05), desirability (*r*_s_ = 0.14, *p* < .05) and aesthetic attachment to place (*r*_s_ = -0.09, *p* < .05). At postcode level, Spearman’s Rho showed a strong significant relationship whereby areas with greater territorial functioning were associated with lower deprivation (*r*_s_ = 0.67, *p* < .01) accounting for 44 % of variance in scores.

**Fig. 1:**
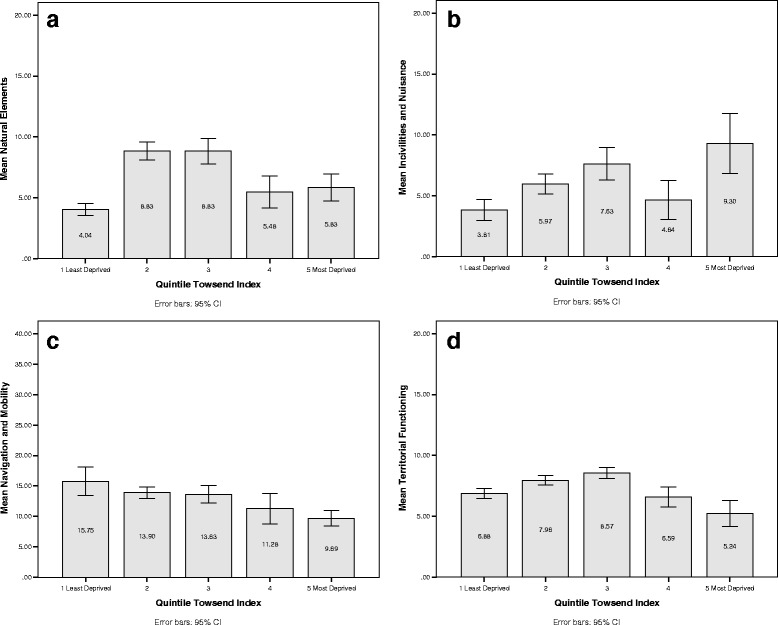
Mean and 95 % confidence intervals of OPERAT domain scores by quintile of the Townsend Index

**Fig. 2:**
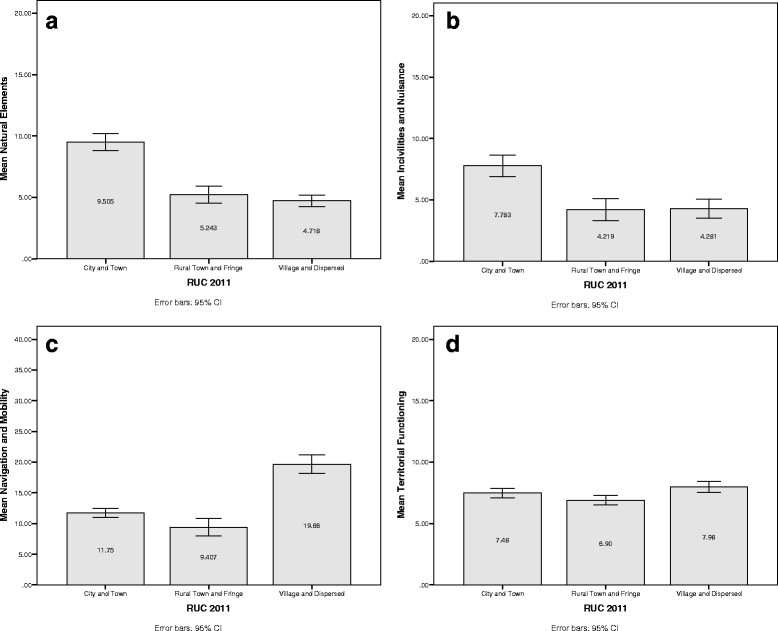
Mean and 95 % confidence intervals of OPERAT domain scores by rural urban categories

The utility of OPERAT was explored through an examination of the variability of the domain scores within neighbourhoods. Figures 1 and 2 show mean scores (and 95 % CI) for each OPERAT domain, whereby neighbourhoods are organised into quintiles of the Townsend Index and settlement type (city and town; rural town and fringe; village and dispersed).

Analysis of variance demonstrated significant differences in the mean scores for each OPERAT domain when organised according to neighbourhood deprivation. The effect of area deprivation on the OPERAT score for incivilities and nuisance was significant (*F*(4, 995) = 9.06, *p* < .001). Post hoc tests (Tukey) demonstrated that mean scores were greater in more deprived areas. With the exception of neighbourhoods classified in the 4th quintile of the Townsend Index, observed incidences of incivilities and nuisance increased with increasing area deprivation. The obverse relationship was discerned between mean scores for navigation and mobility and quintiles of area deprivation (*F*(4, ﻿995) = 17.02, *p* < .001). In this respect, mean scores were lower in more deprived areas. Thus, the observed incidences of barriers to mobility and navigation increased with decreasing area deprivation.

Territorial functioning was significantly associated with area deprivation (*F*(4, 995) = 12.88, *p* < .001). Post hoc analysis indicated that the greatest mean scores for territorial functioning (poorest area upkeep) were observed for neighbourhoods classified in the 1st quintile of the Townsend Index - representing the most deprived areas. However, the relationship between area deprivation and territorial functioning was not linear as the least deprived neighbourhoods (located in the 5th quintile) had similar mean scores to neighbourhoods in the 2nd quintile. Similarly, significant differences in mean scores for natural elements by quintiles of the Townsend Index did not demonstrate a linear relationship (*F*(4, 995) = 23.42, *p* < .001). In this case, post hoc test demonstrated clustering in homogenous subsets whereby, the greatest mean scores (fewest natural elements) were observed in neighbourhoods located in the 2nd and 3nd quintile, and the lowest mean scores (greatest natural elements) were observed in neighbourhoods located in the 1st, 4th and 5th quintiles. The significant but non-linear relationship between the Townsend Index and some of the OPERAT domain scores, demonstrates that area deprivation alone cannot capture all elements of a neighbourhood that may impact on older people.

Analysis of variance demonstrated significant differences in the mean scores for each OPERAT domain when organised according to settlement type. The effect of settlement type on the OPERAT score for natural elements was significant (*F*(2, 497) = 63.80, *p* < .001). The post hoc results showed that city and towns had fewer natural elements than the other two types of areas which comprised a homogenous subset. The same relationship was observed between incivilities and nuisance and settlement type (*F*(2, 497) = 21.32, *p* < .001). Post-hoc tests demonstrated that the least desirable state—a greater level of incivilities and nuisance—was observed in cities and town. Conversely, town and fringe, and village and dispersed settlement types comprised a homogenous subset that had lower mean scores on this domain, representing fewer incivilities and nuisance.

While cities and towns may be considered ‘worse off’ in terms of fewer natural elements, but more incivilities and nuisance, this was not replicated in the analysis of territorial functioning, or navigation and mobility. With regard to navigation and mobility there were significant differences in mean scores between all three settlement types (*F*(2, 497) = 74.15, *p* < .001). Post hoc tests revealed that village and dispersed areas had significantly greater mean scores than city and towns, which in turn had significantly greater mean scores than town and fringe settlements. Thus, older residents in the most rural dispersed areas were more likely to encounter barriers to mobility and navigation.

Analysis of variance demonstrated significant difference between the mean scores for territorial functioning by settlement type (*F*(2, 497) = 4.45, *p* < .05), however, the differences between areas was not so marked as in the other domains. Post-hoc tests demonstrated that mean scores for village and dispersed areas differed significantly from town and fringe with the former having a greater level of territorial functioning than the latter. However, the mean score for city and towns did not differ significantly from the other settlement types. The upkeep and maintenance of gardens and properties in a neighbourhood demonstrates a small amount of variation between settlement types, which is not as pronounced as the differences in territorial functioning observed between neighbourhoods classified in terms of area deprivation.

The inter-rater reliability analysis demonstrated that Krippendorff’s Alpha was greater than the acceptable threshold (0.8) for all items. Raters achieved perfect agreement for 18 items.

## Discussion

In our discussion we compare the four-factor structure of the OPERAT model to the environmental domains described in the introduction, and relate to theories of environmental aesthetics, neighbourhood disorder and environmental stress. We reflect on the involvement of older people in the research process and consider how marginalized groups of older people (e.g. those with a range of physical and cognitive impairments) can co-produce an observational environmental assessment tool. We discuss how participatory approaches to research and robust statistical testing are not mutually exclusive, but can be complementary. We conclude by highlighting some potential uses of the tool for policy and practice, but also point to some limitations that could be addressed by additional research.

### Environmental domains

This article has outlined the development of the Older People’s Environmental Assessment Tool (OPERAT): an observational tool designed to assess the suitability of external residential environments for older people (aged 65+ years) with different physical and cognitive capacities, living in rural or urban areas. Exploratory factor analysis indicated that a four factor model provided an optimal fit to the assessment data collected in 405 postcode areas in Wales. This model included domains capturing *Natural Elements, Incivilities and Nuisance, Territorial Functioning* and *Navigation and Mobility.* Overall, these domains fit well with the theorised domains in the introduction with some exceptions.

We did not identify a separate domain for *Defensible Space.* Items pertaining to this domain were not considered important by older people in the ‘weighting’ survey, nor did the advisory group argue to retain them. Furthermore, we did not identify a separate domain for *Land Use.* However, the item representing an industrial outlook was included in the domain relating to *Territorial Functioning* and is discussed further below. We renamed the *Physical Incivilities* domain *Incivilities and Nuisance,* because while it comprised items expected in the former (litter and dog fouling) it also included items relating to traffic volume and industrial/traffic noise. The factor structure suggested that the items relating to traffic were less to do with safety but were more to do with the auditory and visual impact of passing traffic. Therefore, the traffic items were not subsumed in the hypothesised domain *Accessibility and Safety from Traffic*, instead, we named the remaining factor *Mobility and Navigation*.

The importance that older people ascribed to items and domains in OPERAT resulted in a comprehensive weighting and scoring scheme that was used to compute sub-scale scores and a final score. Statistical testing demonstrated that the domains had internal reliability, construct validity and usability. Subsequently, a tool comprising 17 items^1^ has been developed for use by researchers and lay people to assess external residential environment [see Additional file 3]. Permission for use and instructions for scoring by hand or using SPSS syntax can be obtained from the authors.

#### Environmental aesthetics

The three-item domain *Natural Elements* is related to environmental aesthetics. Our analysis indicated that the presence of natural elements was related to greater aesthetic attachment to place. There were more natural elements found in locations with lower area deprivation, and fewer in cities and towns.

Three categories of biophilic design have been identified: ‘nature in the space’, ‘natural analogues’ and the ‘nature of the space’ [12, 49]. The OPERAT domain *Natural Elements* most closely aligns to ‘nature in the space’ which described the presence and diversity of plant and animal life. Connecting with natural elements can reduce stress, improve mood and self-esteem in the older population [50]. However, the connection to nature may not need to be restricted to physical engagement, ‘viewing’ natural environments (e.g. from a window) may also have a beneficial effect [50]. When functional or cognitive mobility precludes older people from engaging physically with the environment, a view that incorporates natural elements may have a positive impact and satisfy biophilia [51, 52].

Our results indicated that there were fewer natural elements in cities and towns. However, just a few natural elements in an environment can be restorative [50]*.* Biophilic design indicates that both urban and rural environments can be improved in terms of *Natural Elements*. OPERAT provides a tool that can identify areas in which older people would positively benefit from biophilic design interventions.

#### Neighbourhood disorder

The three-item domain relating to *Incivilities and Nuisance* and the five-item domain *Territorial Functioning* are theoretically related to neighbourhood disorder. Our results indicated that greater levels of *Incivilities and Nuisance* were related to feeling unsafe at night and during the day. This is supported by other evidence demonstrating that poor physical environments are associated with increased feelings of insecurity [53]. *Incivilities and Nuisance* were also related to area deprivation, and were more frequently observed in cities and towns than in other settlement types. In areas where older people exhibited *Territorial Functioning* (e.g. gardens and properties were well-maintained), residents reported greater levels of enjoyment, area desirability and aesthetic attachment to place. Moreover, *Territorial Functioning* was more likely in areas of lower deprivation. While improving *Territorial Functioning* may lead to better quality of life, it is more likely that tackling *Incivilities and Nuisance* would lead to reductions in fear of crime for older people in the areas included in our study.

On the whole *Incivilities and Nuisance* refer to public spaces whereas *Territorial Functioning* describes private space*.* For both domains, the least desired state is observed in the most deprived areas where visible neglect by residents, the public and private sector contribute to neighbourhood disorder [54]. *Territorial Functioning* contained an item from the hypothesized *Land Use* domain relating to an industrial, agricultural industrial or commercial outlook. Private non-residential land use impacts on the stewardship of an area, decreases the ability of residents to monitor the space [55], and detracts from the sense of territorial functioning [56].

OPERAT provides a method of assessing neighbourhood disorder represented by *Territorial Functioning* and *Incivilities and Nuisance* which may be particularly important to older people desiring to age in place, or who are constrained from moving because of limited financial or health resources [57].

#### Environmental Stress

The five-item factor that we named *Navigation and Mobility* comprises items that impact on the walkability of a neighbourhood, that in turn can facilitate or hamper spontaneous and intentional social contact. This domain was prioritised by older people who described it as ‘essential’ and all other domains ‘desirable’. It was statistically associated with area enjoyment, perceived desirability of the area and feeling of safety at night. Moreover, the domain was associated with area deprivation although the reverse of what may intuitively be expected: the least desired state (most hazards) were observed in areas of lower deprivation.

*Navigation and Mobility* was also associated with settlement type whereby more barriers to accessibility were observed in remote and rural areas. Most other observational tools are unable to evaluate environmental stress in rural environments because they have been developed in urban areas. Research has tended to focus on urban areas and the fit of the environment to younger people with limited functional abilities [58], with little emphasis on rural accessibility or issues pertinent to older people with cognitive impairment [59].

OPERAT extends environmental assessment to include the needs of older people with functional or cognitive impairment living in urban and rural areas. Road signs may influence wayfinding in those with dementia [60] while adequate street lighting may help older people with visual impairment to navigate the local area [61]. The gradient and quality of the road or pavement may present difficulties for those with mobility problems, and increase the strain for manual wheelchair users [62, 63]. Faced with an environment of hazards and obstructions older people may reduce outdoor activity [3].

Overall, OPERAT can help researchers, planners and older residents identify barriers and hazards that constrain outdoor mobility. It can provide evidence to underpin interventions to facilitate and motivate older adults to move outdoors, thus potentially preventing the development or worsening of disabilities in a spiral of decline [64].

### Participatory approaches to research

Environmental planners have frequently used ‘ableist values’ in design [59] and older people have largely been excluded from contributing to the planning process [65]. OPERAT is comprehensive because we adopted a participatory approach to development: it incorporates the most important and desirable features of the environment for older people. Older people decided on the relative importance of the domains identified in the analysis, and influenced the scoring of the measure. We think that OPERAT may provide a better ‘starting point for understanding pressures on the lives of older people’ (p.601) [65] than ‘idealistic models’ that have been developed without older people, and that have not undergone rigorous psychometric testing.

### Policy and practice implications and limitations

Although OPERAT was developed in Wales, the four-factor model is likely to provide a good fit to data in developed countries. It demonstrates similarities with models that have been theorised in North America, Australia and Europe. While we have used EFA to understand the structure of the data, we did not have a large enough sample to undertake both calibration and validation (with confirmatory factor analysis). Consequently, future research is required to test the fit of the model to other data.

The analysis of the utility of OPERAT indicated that other measures (for example area deprivation) cannot solely account for environmental barriers that older people may encounter. Policy and practice interventions must take into account objective neighbourhood problems (e.g. navigation and mobility, incivilities and nuisance), but also elements which impact on perceptions about the neighbourhood (e.g. natural elements and territorial functioning).

OPERAT has not been designed to map onto the full gamut of domains comprising the World Health Organization’s [6] conceptualisation of age friendly communities but instead is concerned with the physical environment. However, it can identify features of the environment that can support older people to live in familiar neighbourhoods, within their local support network of community relationships [58]. OPERAT may be useful to help in the planning of built environments that are ‘friendly for all ages’—it will help planners to anticipate the impact of the environment on users with different functional and cognitive abilities rather than designing for the ‘able’ [65]. Furthermore, OPERAT can provide older people with tools to understand and critically challenge ‘realities’ in society and equip them with knowledge that can be used to transform these [66]. For example, as part of the Ageing Well in Wales programme in 2016/2017 older people will be coached to become peer-educators in the use of OPERAT, to enable local area audits across Wales.

Large scale epidemiological and cohort studies have been hampered in attempts to model the influence of the environment on health and wellbeing outcomes because of the lack of suitable objective environmental data and coarse resolution of the information that is available (e.g. at the aggregate level of local areas, counties or regions). For researchers, environmental data collected using the OPERAT can be linked to individual data (for example in surveys) and to other extant environmental data. Furthermore, the tool can be used in multiple locations to compile datasets of environmental information that could be consulted by planners to target interventions in particularly deleterious environments, or could be consulted by older adults in planning visits or residential moves to an area. OPERAT has the potential to be developed into a mobile application.

## Conclusions

Very few studies of older adults have directly measured neighbourhood features that may be relevant for understanding the influence of neighbourhoods on health [3], possibly because adequate measurement tools were unavailable. OPERAT fills this gap and statistical testing has shown that the tool has good internal consistency, convergent validity and utility.

Local neighbourhoods can influence the lives of residence through environmental aesthetics, environmental stress and neighbourhood disorder at any age. However, at older ages the risk of being excluded because of functional or cognitive impairment and the pace of environmental change is greater [65]. Therefore it is crucial to identify environmental issues for older people, which OPERAT can provide through observational assessment. The tool has been co-produced with older people with a range of physical and cognitive abilities, residing in various settlement types, ensuring that it is attuned to the diverse needs of the older population.

## Additional files

Additional file 1: Table, Ranks of weighting survey items, mean satisfaction score, median Thurstone scaling score and weight. (PDF 131 kb) Additional file 2: Table, Observational results for assessments items and correlations with validation items. (PDF 323 kb) Additional file 3: Older People’s External Residential Assessment Tool 2016. (PDF 139 kb)

## Abbreviations

ANOVA: Analysis of variance
CFI: Comparative fit indices
EAG: Expert Advisory Group
EFA: Exploratory factor analysis
EM: Expectation-maximization
LSOA: Lower Super Output Area
NeDeCC: Neighbourhood Design Characteristics Checklist
NIfETY: Neighbourhood Inventory for Environmental Typology
NOC: Healthy Environments Partnership Neighbourhood Observational Checklist
OPERAT: Older People’s External Residential Assessment Tool
REAT: Residential External Assessment Tool
RMSEA: Root mean square error of approximation
SPACES: Systematic Pedestrian and Cycling Environmental Scan
TLI: Tucker Lewis Index
UK: United Kingdom

## Footnotes

1 There were 16 items included in the EFA model. However, one item has been split into two questions for the tool (street lighting and unlit alleys). These are subsequently recoded during scoring.
