# Supplementary material for: Older People’s External Residential Assessment Tool (OPERAT): a complementary participatory and metric approach to the development of an observational environmental measure
Source: BMC Public Health. 2016 Sep 29;16:1022. doi: 10.1186/s12889-016-3681-x (PMC5041557; doi:10.1186/s12889-016-3681-x)
Supplement: Additional file 1: — Table, Ranks of weighting survey items, mean satisfaction score, median Thurstone scaling score and weight. (PDF 131 kb) [file 12889_2016_3681_MOESM1_ESM.pdf]

Additional File 1.

Table: Ranks of weighting survey items, mean satisfaction score, median Thurstone scaling score and weight

Rank	Item	Mean (SD)[a]	Median[b]	Weight
1	A view of green space (e.g. fields/forests)	4.17 (1.17)	9	4
2	Sounds of nature (e.g. birdsong, water)	4.14 (1.22)	9	4
3	A well-maintained road (e.g. no pots holes/cracks)	4.13 (1.02)	8	3
4	Dog fouling	4.13 (1.22)	1	-4
5	A poorly maintained road	3.99 (1.17)	2	-3
6	Litter	3.99 (1.23)	2	-3
7	A well-maintained pavement	3.96 (1.24)	8	3
8	A poorly maintained pavement	3.95 (1.30)	2	-1
9	Burned out/abandoned properties	3.93 (1.47)	2	-3
10	Well maintained property exteriors	3.93 (1.53)	8	3
11	Presence of a pavement	3.92 (1.25)	8	3
12	Loud traffic or industrial noises	3.87 (1.36)	2	-3
13	Broken glass	3.86 (1.44)	2	-3
14	Street lighting	3.86 (1.30)	8	3
15	Low traffic level	3.85 (1.27)	8	3
16	Well maintained gardens	3.85 (1.22)	8	3
17	Poorly maintained property exteriors	3.84 (1.22)	2	-3
18	Vandalism	3.83 (1.51)	2	-3
19	High traffic level	3.82 (1.34)	2	-3
20	Poorly maintained gardens	3.74 (1.20)	2	-3
21	An industrial view (e.g. steel works/factories)	3.68 (1.49)	2	-3
22	Concrete or tarmac pavement	3.64 (1.33)	8	3
23	A wide pavement	3.64 (1.32)	8	3
24	Private trees/hedges/foliage	3.58 (1.19)	8	3

25	Litter bins	3.57 (1.28)	8	3
26	Cars parked on the pavement	3.55 (1.27)	2	-3
27	Gritting bins for cold conditions	3.54 (1.27)	7	2
28	Poorly maintained public buildings (e.g. libraries)	3.52 (1.41)	2	-3
29	Graffiti	3.52 (1.52)	2	-3
30	Well maintained public buildings	3.52 (1.38)	8	3
31	Traffic calming measures (e.g. speed bumps/chicanes)	3.49 (1.37)	5	0
32	Level pavement/road	3.47 (1.25)	7	2
33	Continuous pavement	3.45 (1.36)	7	2
34	Public trees/hedges/foliage	3.44 (1.26)	7	2
35	Public grass or verges	3.43 (1.25)	7	2
36	Mainly non-restricted parking	3.43 (1.34)	5	0
37	A sea view	3.43 (1.50)	8	3
38	A narrow pavement	3.43 (1.32)	3	-2
39	House numbers or names on properties	3.42 (1.30)	8	3
40	Public or private decoration (e.g. hanging baskets)	3.84 (1.27)	8	3
41	Mainly residents only parking	3.34 (1.39)	7	2
42	Bus shelter	3.34 (1.44)	7	2
43	Well maintained bus shelter	3.31 (1.43)	7	2
44	Clear and easy to read road name sign	3.30 (1.41)	7	2
45	Bus stop	3.29 (1.44)	7	2
46	White bar markings (to stop parking in front of driveways)	3.25 (1.40)	7	2
47	Walls or buildings which block out the light	3.25 (1.50)	3	-2
48	Unlit alleyways	3.25 (1.53)	3	-2
49	Poorly maintained bus shelter	3.25 (1.47)	3	-2
50	Speed cameras	3.24 (1.40)	5	0
51	An agricultural industrial view (e.g. farmyard machinery)	3.23 (1.43)	3	-2

52	Seating in bus shelter	3.21 (1.40)	7	2
53	Dark spots between lighting	3.19 (1.40)	3	-2
54	Benches or other seating	3.18 (1.38)	7	2
55	Road name sign	3.17 (1.44)	7	2
56	Cobble pavement	3.11 (1.49)	3	-2
57	Steep slope to pavement/road	3.10 (1.35)	3	-2
58	A residential view (e.g. housing estates/ cul-de-sac)	3.10 (1.32)	5	0
59	Dropped kerbs at crossings	3.08 (1.42)	7	2
60	Tree cover/canopy in the street	3.06 (1.37)	5	0
61	Mid-pavement lamp posts	3.06 (1.34)	3	-2
62	Low overhanging trees	3.06 (1.33)	3	-2
63	Public toilets	3.05 (1.50)	5	0
64	A commercial view (e.g. a high street)	3.05 (1.42)	3	-2
65	Man-hole covers on the pavement	3.04 (1.40)	3	-2
66	Flagstone pavement	3.04 (1.43)	5	0
67	Pedestrian crossings	3.01 (1.46)	6	1
68	CCTV cameras	2.97 (1.38)	6	1
69	Neighbourhood watch signs	2.96 (1.34)	7	2
70	Mainly detached properties	2.89 (1.38)	7	2
71	Steps on the pavement	2.88 (1.38)	4	-1
72	Fences, gates, walls or hedges outside properties	2.82 (1.41)	6	1
73	Other markers to help navigation	2.81 (1.36)	6	1
74	Mainly flats	2.79 (1.37)	3	-2
75	Security bars on windows	2.79 (1.49)	4	-1
76	Railings/other assistance	2.78 (1.35)	6	1
77	Lighting from other sources (e.g. overnight businesses)	2.75 (1.50)	4	-1
78	Mainly agricultural/farming buildings	2.72 (1.35)	5	0
79	Tactile paving on crossings	2.69 (1.34)	6	1

80	Windows overlooking the street	2.64 (1.38)	6	1
81	Mainly semi-detached properties	2.62 (1.28)	6	1
82	Mainly terraced properties	2.62 (1.27)	5	0
83	For sale signs/vacant properties	2.52 (1.27)	4	-1
84	Colour of the pavement to contrast with kerb and road	2.51 (1.30)	6	1

[a] Mean satisfaction score: range 1-5

[b] Median after Thurstone Scaling: range 1-
