# Supplementary material for: Older People’s External Residential Assessment Tool (OPERAT): a complementary participatory and metric approach to the development of an observational environmental measure
Source: BMC Public Health. 2016 Sep 29;16:1022. doi: 10.1186/s12889-016-3681-x (PMC5041557; doi:10.1186/s12889-016-3681-x)
Supplement: Additional file 2: — Table, Observational results for assessments items and correlations with validation items. (PDF 323 kb) [file 12889_2016_3681_MOESM2_ESM.pdf]

Additional File 2

Table: Observational results for assessments items and correlations with validation items

**OPERAT item**	**% (yes)**	**Validation item**[c]	**Kendall's Tau b**	**Tested in EFA**
Are there gritting bins for cold conditions?	16.0	There are enough gritting bins	-0.09*	✓
Are there litter bins?	16.0	There are enough litter bins	-0.03*	✓
Are there road name signs?	69.6	The road name signs are clear and easy to read	-0.31	‡
Are the road signs clear and easy to read?	69.4	The road name signs are clear and easy to read	-0.03	‡
Are there traffic calming measures?	4.4	There are enough traffic calming measures	-0.04	
Are there loud traffic or industrial noises?	27.4	Loud traffic or industrial noises are a problem	-0.24**	✓
Public grass or verges?	46.4	There are not enough green areas or trees	-0.14**	✓
Sounds of nature (e.g. birdsong, water)?	83.0	Sounds of nature can be heard	-0.17**	✓
Are there street lights?	81.5	A lack of street lighting is a problem	-0.01	‡
Are there any unlit alleyways?	35.6	Unlit alleyways are a problem	-0.07	‡

Is there a bus stop?	9.6	The proximity of a bus stop is a problem	0.05	
Is there a bus shelter?	5.7	The quality of a bus stop is a problem	0.08	
Is there seating in the bus shelter?	5.4	The quality of a bus stop is a problem	0.08	
Is there a pavement?	72.6	The lack of a pavement is a problem	0.15**	‡
Is the pavement continuous?	61.0	The lack of a pavement is a problem	0.21**	‡
Main outlook				✓
Residential	50.1	This is a desirable place to live	0.10*	
Green or sea	45.7	This is a desirable place to live	-.010*	
Industrial, agricultural or commercial	4.2	This is a desirable place to live	-0.01	
Name/Number on property?	87.8	The properties names or numbers are clear and easy to read	-0.09**	✓
Trees in garden?	38.1	There are not enough green areas or trees	0.17**	✓
External beautification?	62.4	The area is nicely kept by its residents	-0.12**	✓

%

How well maintained are public buildings?		Public buildings are well maintained	0.01	
N/A	86.7			
Poorly	1.5			
Moderately	2.7			
Well	9.1			
What is the nature of parking on the street?				✓
Residents Only Parking	27.2	There are enough parking spaces for residents and visitors	-0.29**	
Non-Restricted	22.0	There are enough parking spaces for residents and visitors around here	0.15**	
Mixture	50.9	There are enough parking spaces for residents and visitors	0.18**	
How well maintained is the bus shelter?				
N/A	92.6	The quality of the bus stop is a problem	-0.08	
Poorly	0.2			
Moderately	2.0	The quality of a bus stop is a problem	0.09*	

Well	5.2	The quality of a bus stop is a problem	0.04	
How well is pavement maintained?				‡
N/A	27.2	The condition of the pavements is a problem	0.01	
Poorly	2.7	The condition of the pavements is a problem	-0.04	
Moderately	60.0	The condition of the pavements is a problem	-0.08	
Well	10.1	The condition of the pavements is a problem	0.12**	
What is the type of pavement?				
N/A	27.4	The condition of the pavements is a problem	0.01	
Tarmac	70.4	The condition of the pavements is a problem	-0.01	
Flagstone	2.2	The condition of the pavements is a problem	-0.01	
Cobble	0.0			
How wide is the pavement? *(%)*				‡
N/A	26.9	The condition of the pavements is a problem	0.01	
Narrow	8.1	The condition of the pavements is a problem	-0.01	
Medium	53.3	The condition of the pavements is a problem	-0.03	
Wide	11.6	The condition of the pavements is a problem	0.04	

How well is road maintained?		The condition of the road is a problem	0.18**	✓
Poorly (or no road in postcode)	5.8			
Moderately	55.0			
Well	39.2			
Pavement/road gradient?		The incline of the road is a problem	0.22***	✓
Flat	51.9			
Medium (slight incline)	30.9			
Steep (substantial incline)	17.3			
Garden maintenance?				✓
N/A	9.9			
Poor	8.7	The area is nicely kept by its residents	0.02	
Medium	40.3	The area is nicely kept by its residents	-0.01	
Well	41.1	The area is nicely kept by its residents	-0.18**	
Property maintenance (as a % of all properties)				✓
Poor	5.7	The area is nicely kept by its residents	0.10**	

Moderate	44.4	The area is nicely kept by its residents	0.07*	
Well	49.9	The area is nicely kept by its residents	-0.18**	
	**Mean (SD)**			
Number of benches/other seating?	0.17 (0.65)	There are enough benches and places to sit	-.10*	‡
Number of cars parked on the pavement?	0.68 (1.56)	Cars parking on the pavement is a problem	-0.10*	‡
Number of instances of vandalism?	0.01 (0.11)	Vandalism is a problem	-0.08*	‡
Number of instances of graffiti?	0.02 (0.19)	Graffiti is a problem	-0.07	‡
Number of instances of dog fouling?	0.05 (0.29)	Dog fouling is a problem	0.03	‡
Number of instances of littering?	0.36 (0.83)	Litter is a problem	-0.12**	‡
Number of instances of broken glass (on the ground)?	0.00 (0.05)	Broken glass on the ground is a problem	-0.04	‡
Number of burned out/abandoned properties?	0.06 (0.28)	Burned out or abandoned properties are a problem	-0.04	
Number of public trees?	1.83 (4.00)	There are not enough green areas or trees	-0.03	
Number of vehicles passed during assessment?	3.41 (7.17)	There is a high volume of traffic	-0.30**	‡

Number of walls or buildings blocking the light in the street?	0.32 (0.61)	Walls or buildings which obstruct the light are a problem	-0.05	‡

* p<.05 **p<.005 *** p<.001

✓ Tested in EFA models

‡ Tested in EFA models as modified variable

[c] Each item specified ‘around here’
