# Supplementary material for: Older People’s External Residential Assessment Tool (OPERAT): a complementary participatory and metric approach to the development of an observational environmental measure
Source: BMC Public Health. 2016 Sep 29;16:1022. doi: 10.1186/s12889-016-3681-x (PMC5041557; doi:10.1186/s12889-016-3681-x)
Supplement: Additional file 3: — Older People’s External Residential Assessment Tool 2016. (PDF 139 kb) [file 12889_2016_3681_MOESM3_ESM.pdf]

Older People's External Residential Assessment Tool 2016

Postcode			Number of properties	

Date		Time of assessment		Duration of assessment	

Street Level Observations

Tick yes or no for items 1-8	YES	NO
1. Is there public grass or verges?		
2. Are there sounds of nature (e.g. birdsong, water)?		
3. Are there clear and easy to read road name signs?		
4. Are there street lights?		
5. Are there any unlit alleyways?		
6. Are there instances of littering, dog fouling or broken glass?		
7. Are there loud traffic or industrial noises?		

Tick a single box which corresponds to the response for items 8-13

8. Approximate number of vehicles that drove past during assessment?

- [ ] NONE
- [ ] ONE TO ELEVEN
- [ ] TWELVE OR MORE

9. What is the nature of parking on the street?

- [ ] RESIDENTS ONLY
- [ ] NOT RESIDENTS ONLY

10. Is there a continuous pavement, that is wide enough for 2 people or a wheelchair and is well maintained

- [ ] NO PAVEMENT
- [ ] YES, BUT NOT CONTINUOUS, NARROW OR NOT WELL MAINTAINED
- [ ] YES, CONTINUOUS, WIDE/MODERATELY WIDE, WELL MAINTAINED

11. How steep is the pavement and/or road?

- [ ] FLAT:
- [ ] MEDIUM: Slight incline, not troublesome to walk up
- [ ] STEEP: Substantial incline, taxing to walk up

12. How well is the road maintained?

- [ ] WELL: Good condition, no maintenance required
- [ ] MODERATELY: Only minor repairs required
- [ ] POORLY: Lots of pot holes, trip risks, no evidence of repair

13. What is the main outlook?

- [ ] RESIDENTIAL
- [ ] GREEN OR SEA
- [ ] AGRICULTURAL INDUSTRIAL, INDUSTRIAL OR COMMERCIAL

Property Level Observations

Tick response for items 14-17 for each property listed in the 1st column. Count and total the ticks in the highlighted columns

Name or number of property	14. Are there trees in the garden?		15. Is there any external beautification?			16. How well maintained is the garden/front yard?				17. How well maintained is the outside of the property		
	YES	NO	YES	NO	N/A	WELL	MOD	POOR	N/A	WELL	MOD	POOR
**TOTAL**												
